# Supplementary figures and images for: MCHelper automatically curates transposable element libraries across eukaryotic species
Source: Genome Res. 2024 Dec;34(12):2256–68. doi: 10.1101/gr.278821.123 (PMC11694758; doi:10.1101/gr.278821.123)

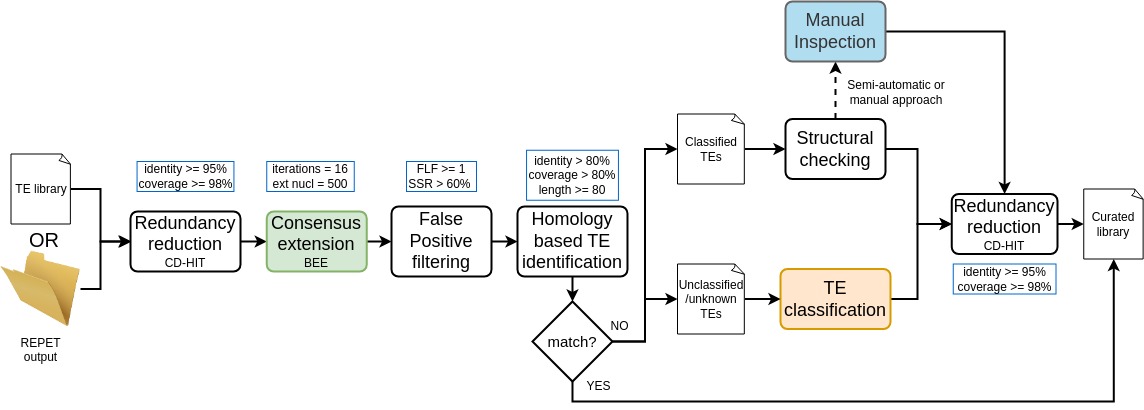

Supplement: Supplement 1 [file Supplemental_Code.zip › MCHelper-main/MCHelper_Flow.jpg]

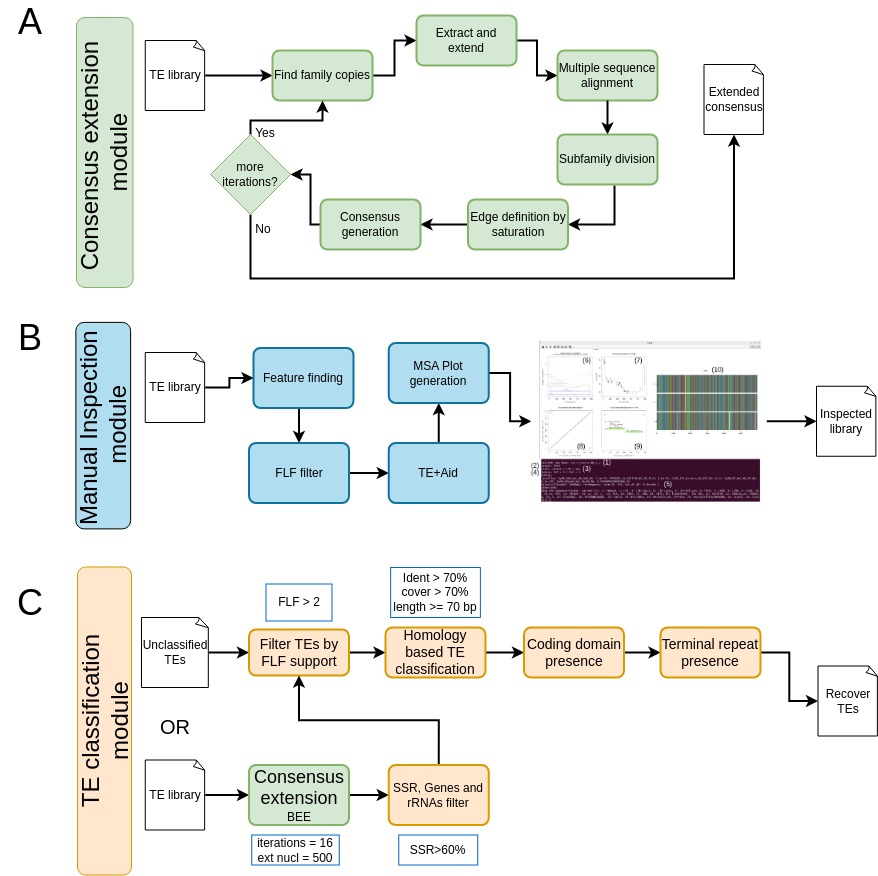

Supplement: Supplement 1 [file Supplemental_Code.zip › MCHelper-main/MCHelper_modules_Flow.jpg]

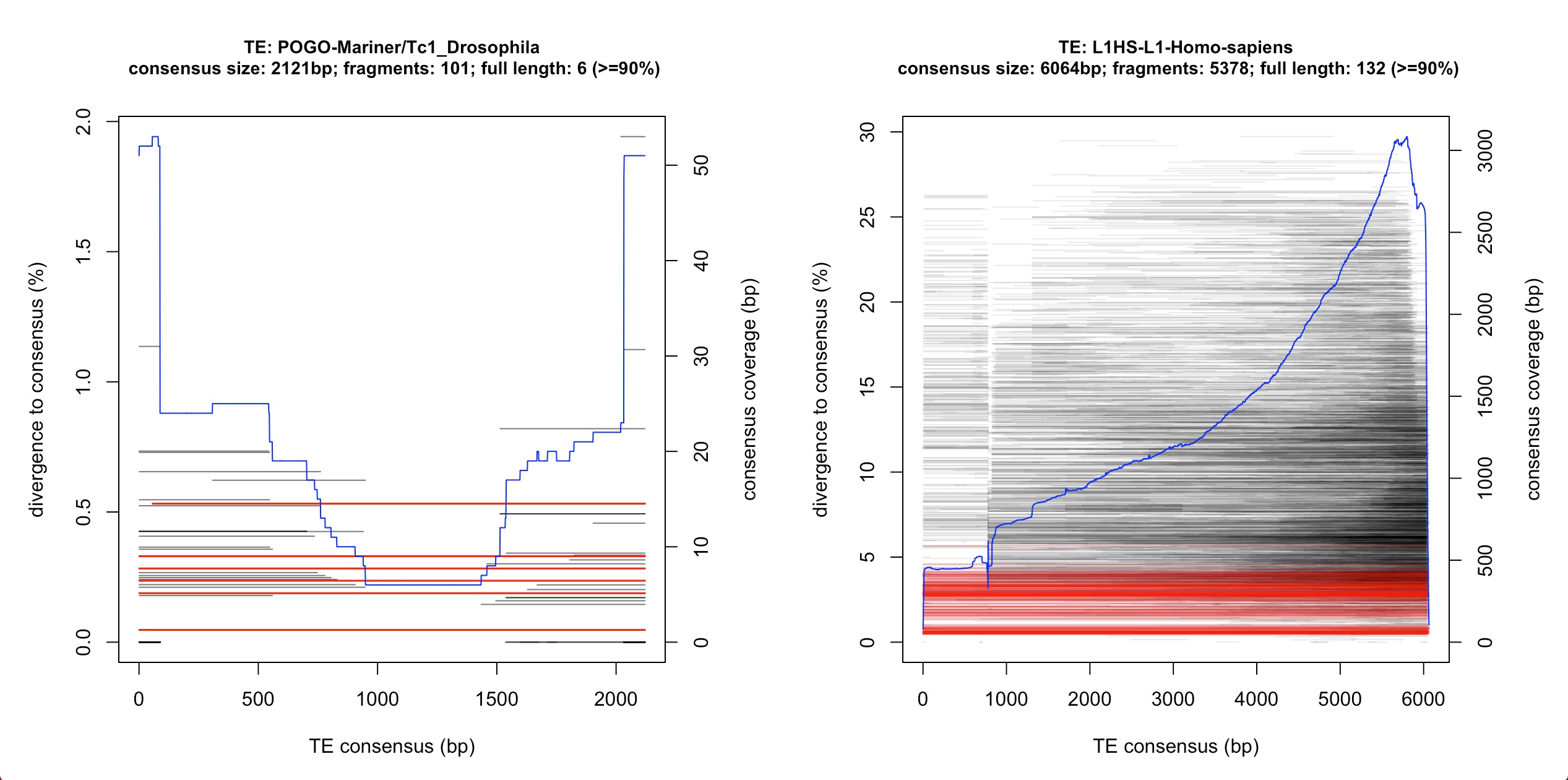

Supplement: Supplement 1 [file Supplemental_Code.zip › MCHelper-main/tools/TE-Aid-master/Example/cons2gen.jpeg]

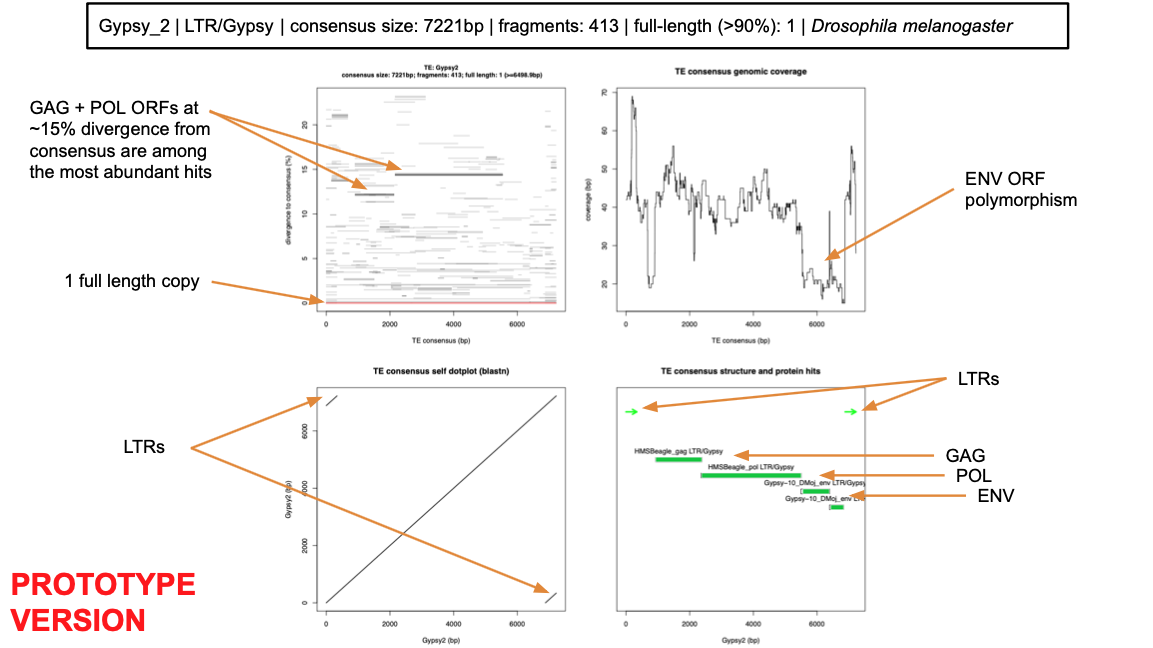

Supplement: Supplement 1 [file Supplemental_Code.zip › MCHelper-main/tools/TE-Aid-master/Example/Gypsy2.TEaid.png]

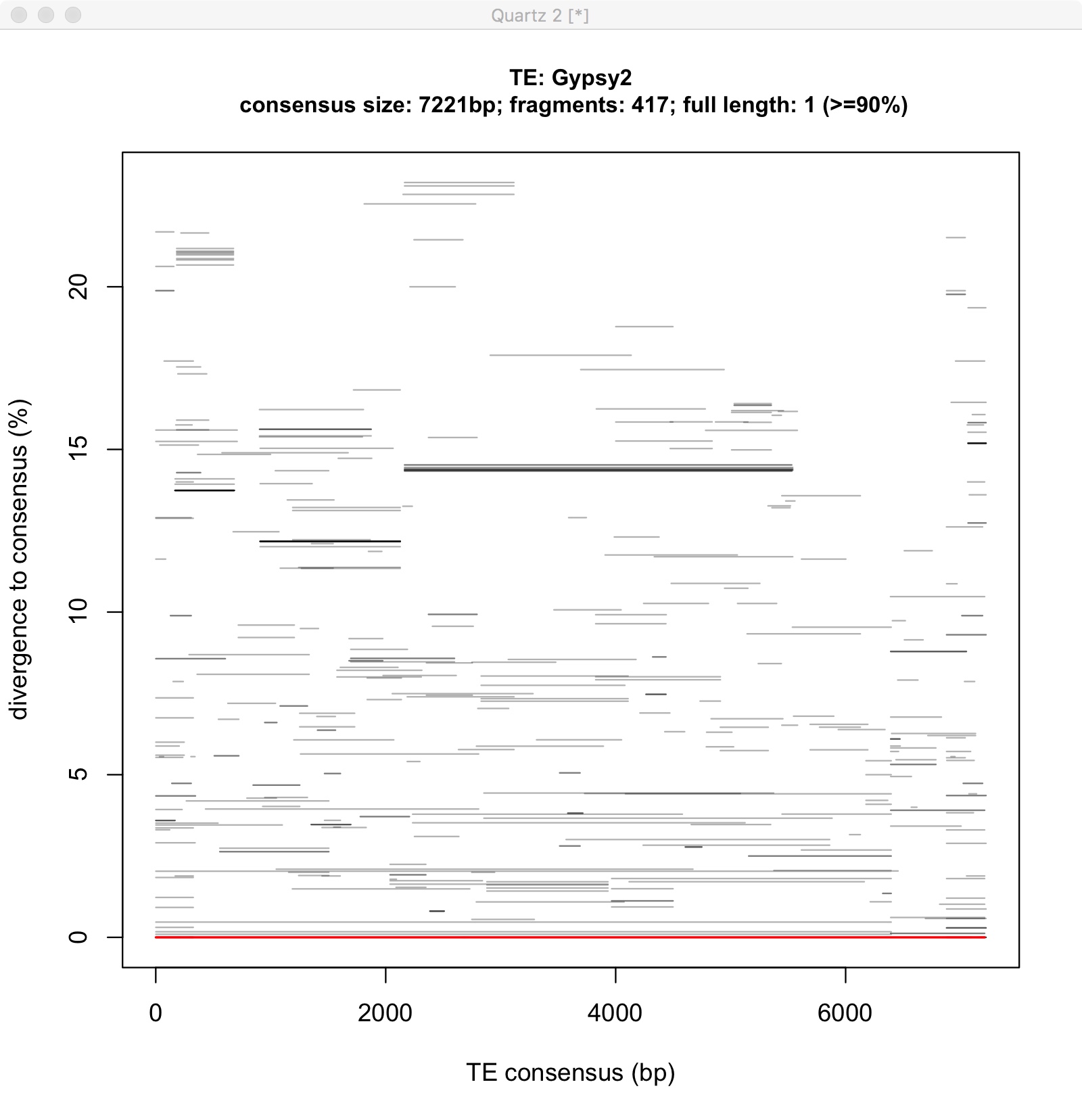

Supplement: Supplement 1 [file Supplemental_Code.zip › MCHelper-main/tools/TE-Aid-master/Example/Gypsy_example.jpeg]

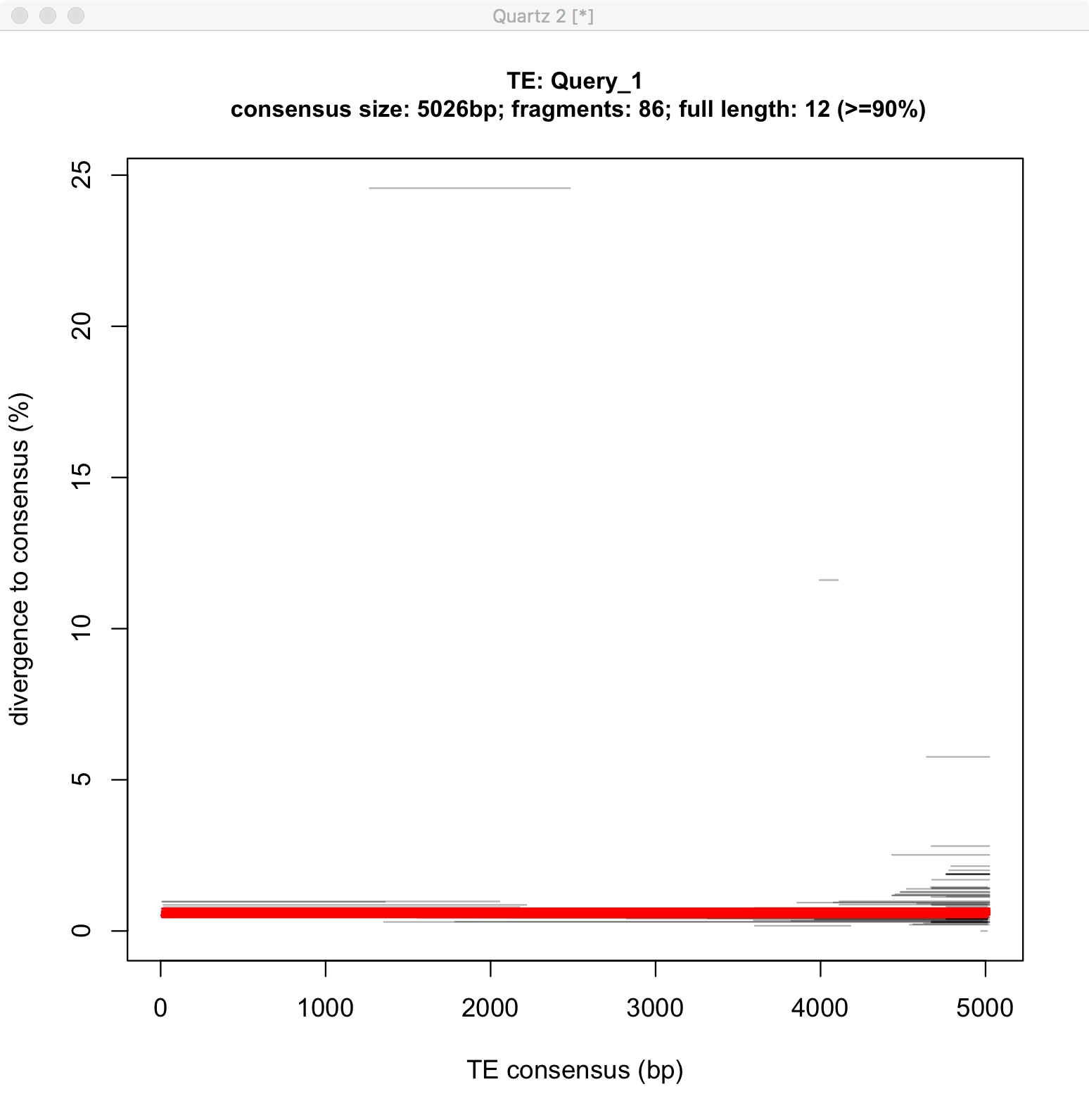

Supplement: Supplement 1 [file Supplemental_Code.zip › MCHelper-main/tools/TE-Aid-master/Example/Jockey-1.jpeg]

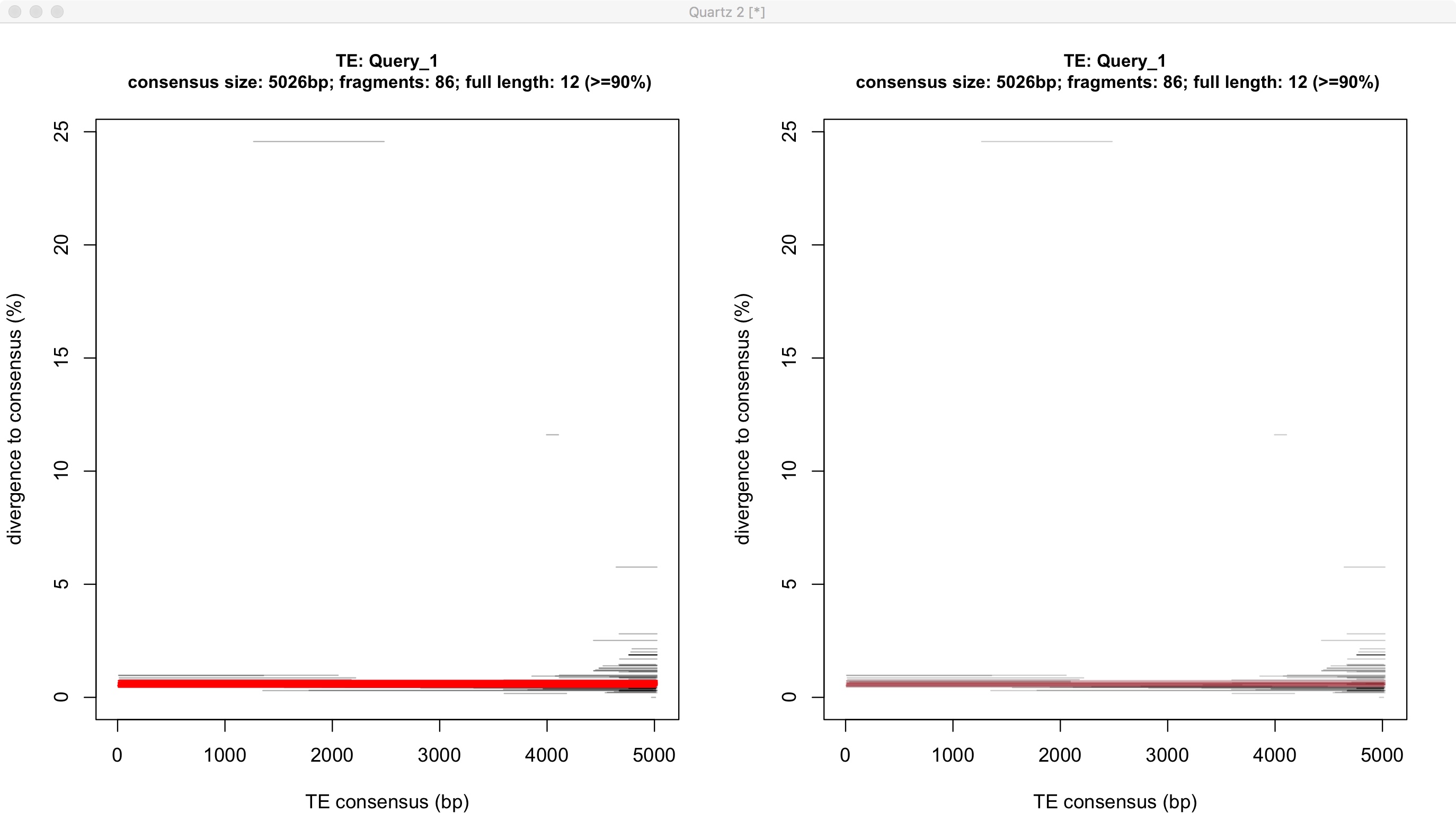

Supplement: Supplement 1 [file Supplemental_Code.zip › MCHelper-main/tools/TE-Aid-master/Example/Jockey-2.jpeg]

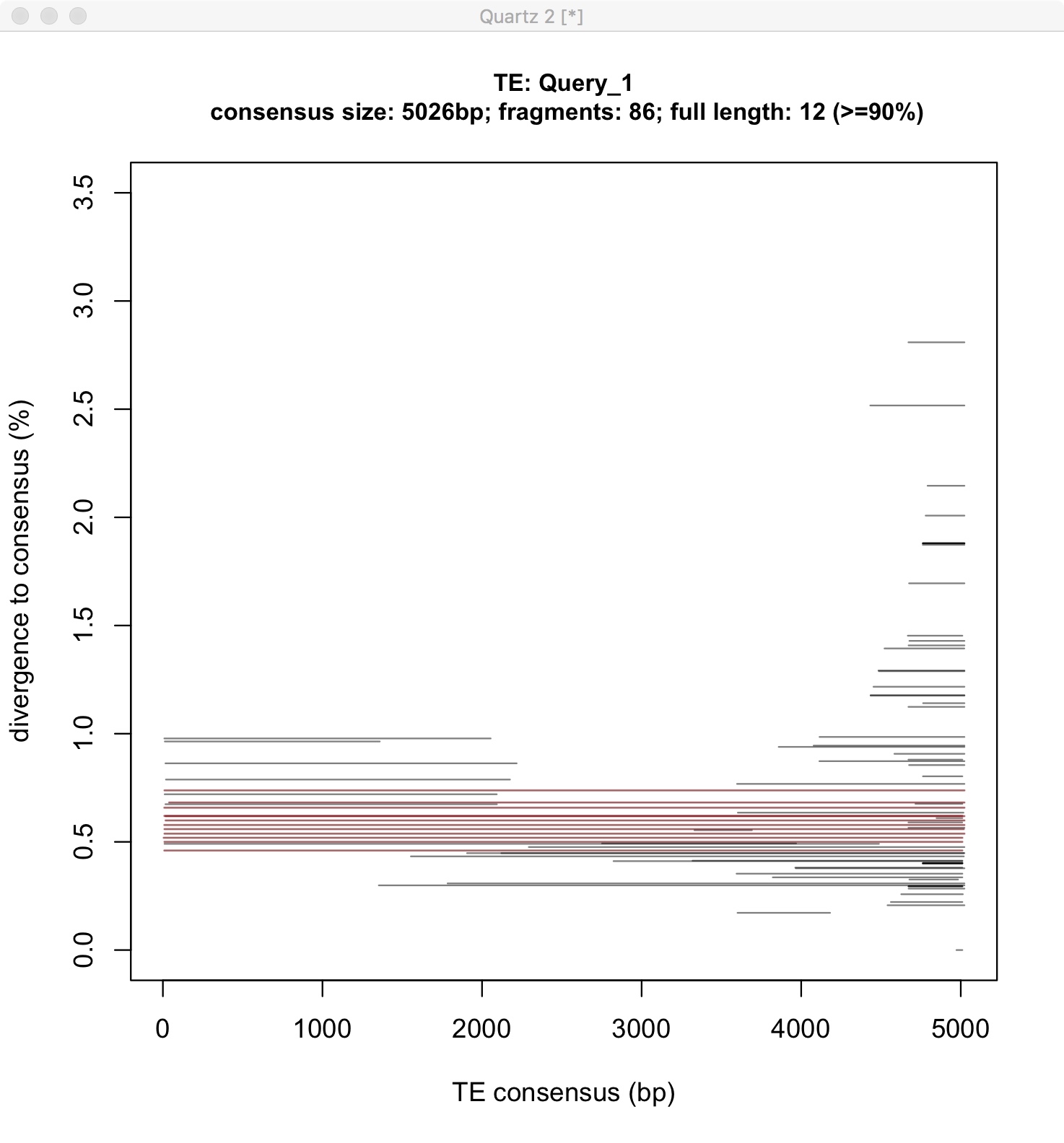

Supplement: Supplement 1 [file Supplemental_Code.zip › MCHelper-main/tools/TE-Aid-master/Example/Jockey-3.jpeg]

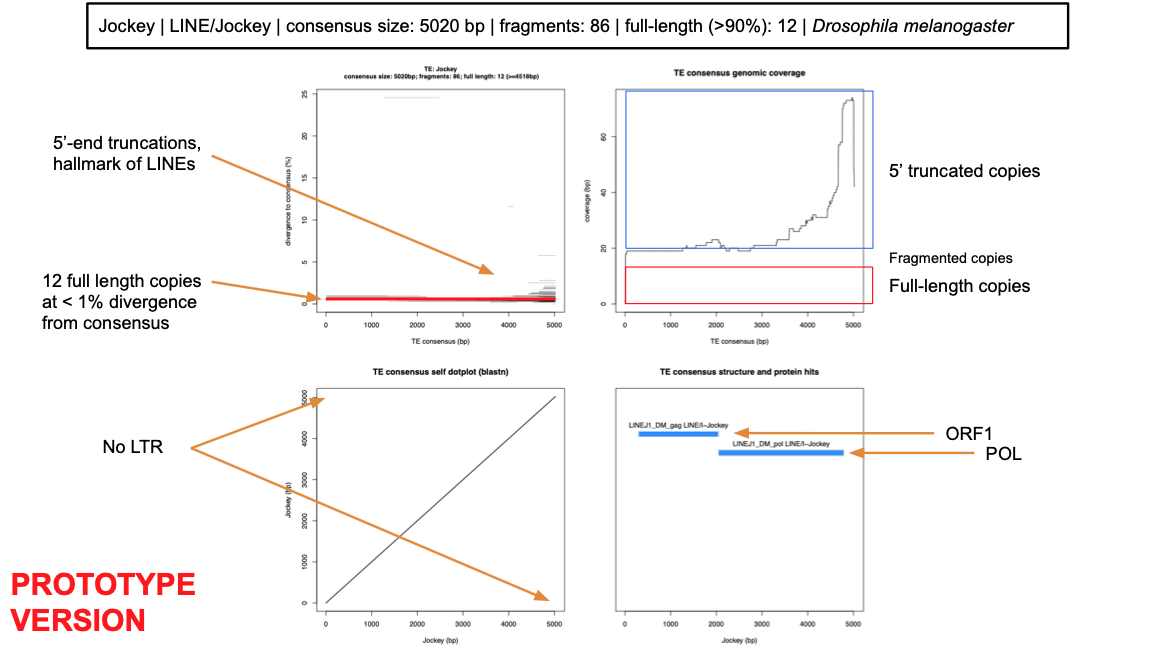

Supplement: Supplement 1 [file Supplemental_Code.zip › MCHelper-main/tools/TE-Aid-master/Example/Jockey.TEaid.png]

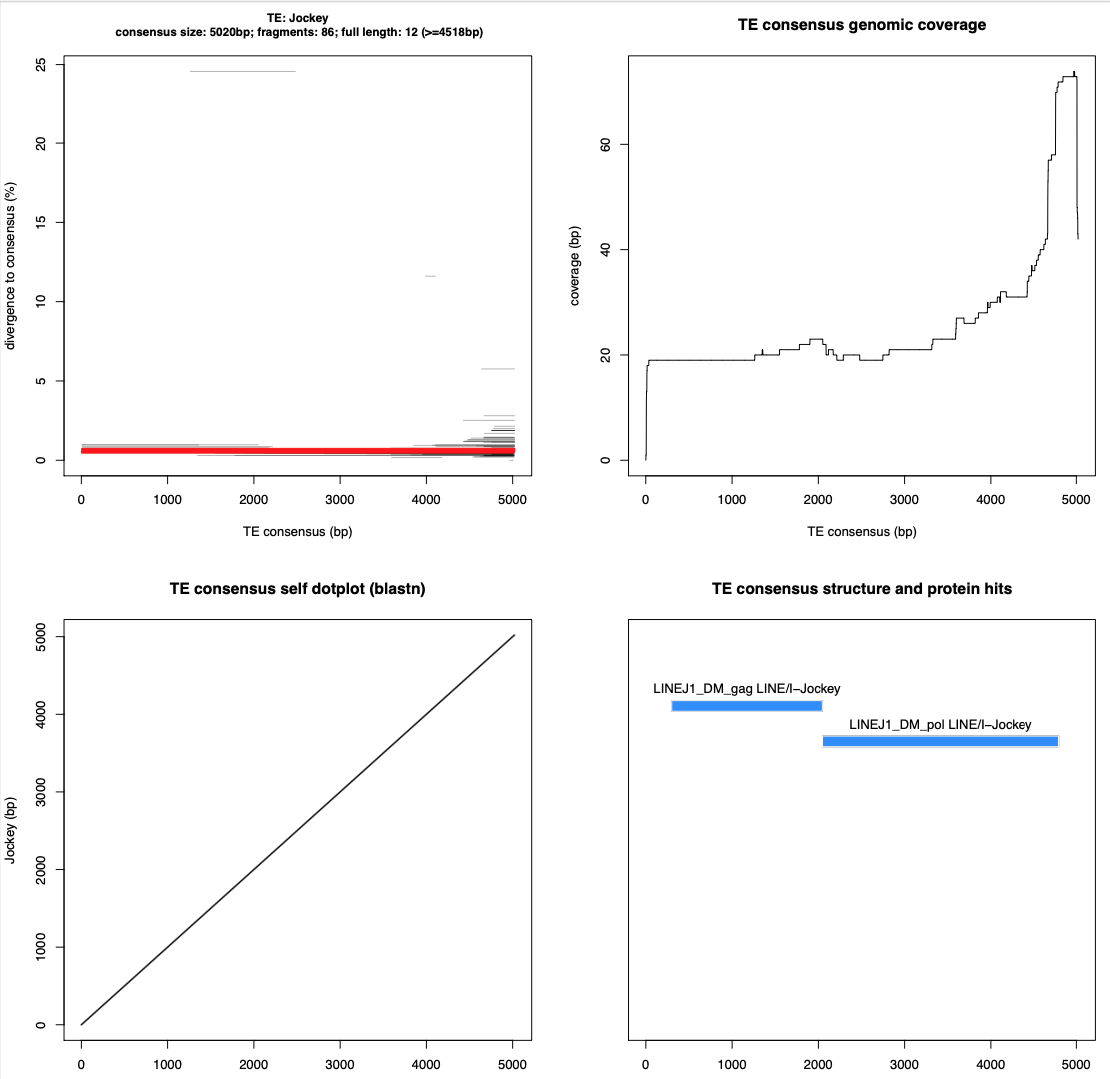

Supplement: Supplement 1 [file Supplemental_Code.zip › MCHelper-main/tools/TE-Aid-master/Example/Jockey_new.jpeg]

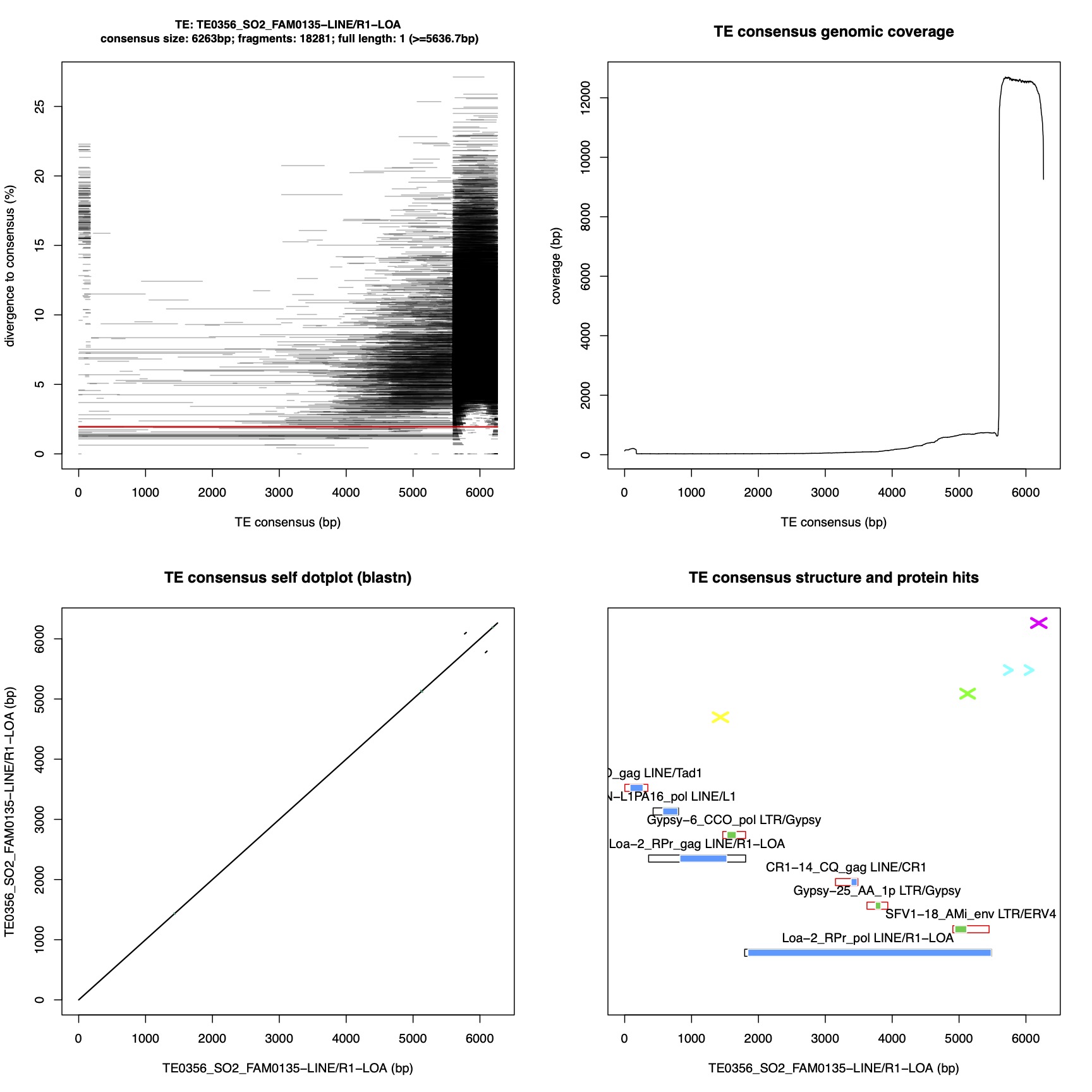

Supplement: Supplement 1 [file Supplemental_Code.zip › MCHelper-main/tools/TE-Aid-master/Example/LINE.1.fasta.c2g.jpg]

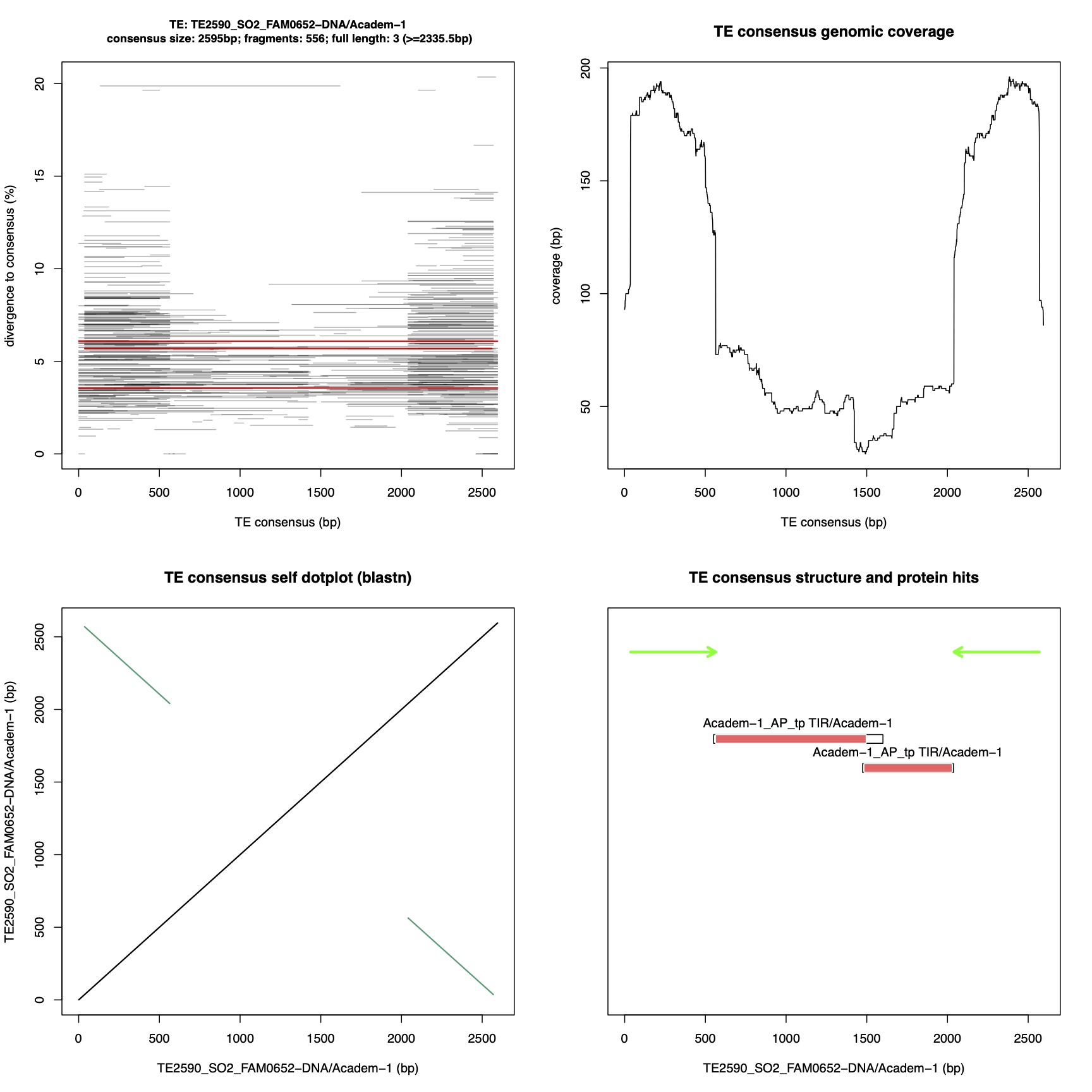

Supplement: Supplement 1 [file Supplemental_Code.zip › MCHelper-main/tools/TE-Aid-master/Example/TE1.jpeg]
